# Exploring Teachers' Attitudes toward the Management of Type 1 Diabetes: A Qualitative Study

**DOI:** 10.1155/2023/6607310

**Published:** 2023-09-28

**Authors:** Maria Dora Horvath, Orsolya Papp-Zipernovszky, Zsanett Tesch, Norbert Buzas

**Affiliations:** ^1^Department of Health Economics, Albert Szent-Györgyi Medical School, University of Szeged, Szeged, Hungary; ^2^Department of Personality, Clinical and Health Psychology, Institute of Psychology, University of Szeged, Szeged 6720, Hungary; ^3^Department of Personality and Health Psychology, Institute of Psychology, Faculty of Education and Psychology, Eötvös Loránd University, Budapest 1064, Hungary; ^4^Department of Health Sciences and Health Management, Faculty of Health Sciences and Social Studies, University of Szeged, Szeged, Hungary

## Abstract

This study aimed to explore the attitudes of teachers toward type 1 diabetes (T1D) and its management. Teachers working in kindergartens and schools (*N* = 30) participated in audio-recorded, semi-structured interviews (three focus groups and 20 individual interviews) that were transcribed and analyzed using thematic analysis. We used the theory of the three components of attitude as a framework for the analysis. The three components of attitude emerged during the analysis: knowledge, positive and negative emotions, approaches, and opinions toward diabetes and its management and behavior. The main theme of knowledge included knowledge about diabetes in general and its management. Besides medical treatment, alternative treatment possibilities were mentioned by the participants. The affective component revealed empathy, integrating, and segregating approaches toward children living with diabetes. The behavior component revealed how teachers contribute to the care and integration of children with diabetes in schools. They support children with diabetes by the virtue of their profession. For example, they teach them health awareness and support their integration through peer education and sensitization. The findings indicated that, in addition to diabetes management tasks, teachers could help children with T1D by tutoring them and their peers about health awareness and T1D acceptance.

## 1. Introduction

The management of type 1 diabetes (T1D) in children requires constant monitoring and care [1]. This includes various tasks, such as monitoring blood glucose levels, nutrition, taking care of the insulin regimen, and paying attention to the amount of physical activity the child undertakes. A basic level of specific knowledge and skills is necessary for the proper treatment of T1D, as it is rather complex and involves invasive procedures such as blood glucose monitoring and insulin insertion [2]. These tasks are challenging for children, especially when it comes to modern insulin regimens [3]. Parents, as the primary caregivers of children with T1D, also face many unique challenges concerning everyday responsibilities, continuous supervision, and caregiving [4]. Hence, children with T1D and their parents might often need help from school staff or teachers.

However, children with T1D face further challenges and difficulties in school and kindergarten, which mostly come from the lack of informed and trained staff, the absence of nurses, or the lack of diabetes care policies in schools [5]. There may be some institutions where professional medical help (e.g., school nurse) is available to support children with T1D in their regimen tasks. For example, the MOCHA project found that across five European countries, Norway and Estonia report around 1.4 school nurses on average per 1000 pupils, Finland 1.2, and Iceland 0.9 [6].

Deficient diabetes management in schools may cause several troublesome consequences, such as absenteeism, stress or depression, poor performance, and low quality of life [5]. Children with T1D who make negative attributions of teachers' reactions regarding self-care efforts have more difficulties related to adherence in school situations, and they experience more stress [7]. They may feel unconfident about their condition in the school setting and avoid performing management tasks to evade undesirable attention and notions of feeling “different” from peers [8].

Several schools and kindergartens are not able to support children with T1D effectively. As the Dawn Youth Study—a fact-finding survey executed in 24 countries—demonstrated diabetes laws and regulations, the availability of nurses and diabetes training was inadequate or deficient in most of the schools investigated [9]. Tolbert [10] reviewed 11 articles focusing on the improvement of diabetes management in schools. According to his findings, the school staff often provide direct support for students with T1D. He notes that improvement would be necessary in the following areas: communication, after-school support, education of staff and peers, school nurse availability, and lunch choices. In a more recent Spanish study, 43% of the teachers had taught children with diabetes, but only 0.8% had received specific training in diabetes care [11].

Holmström et al. [12] investigated school personnel's experience of caring for youth with T1D. They conducted interviews with 24 school personnel (having experience with youth aged 6–18 years). According to their results, school personnel characterized the experience as “being facilitators in a challenging context” (p. 116). They reported feeling uncertain and incapable as they dealt with never-ending, unclear responsibilities. The teachers had to find their own way to cope with these difficulties and challenges. They found strategies to support self-care and established trusting relationships with the youth and their parents. This study also highlighted the need for educating school personnel and nurse specialists.

Diabetes management can be most effective by maintaining a partnership among students, parents, school nurses (if they are available), health care providers, teachers, and other related school personnel (transportation, food service employees, and administrators) [13]. Teachers have an important role in the management of diabetes in schools and kindergartens. If the child is too young for independent management, it may be necessary to help with specific aspects of management. If the child is already self-managed, the teacher's approach and attitude of the management tasks can be essential. Several studies have investigated teachers' perceptions and attitudes toward diabetes care [11, 14–16]. These studies used quantitative tools and got divergent results (e.g., for willingness to participate in management), which calls for further investigation of the subject. A qualitative study allows us to explore the issue in more depth and may shed light on factors that are less likely to be revealed by quantitative tools.

Allport [17] defines attitude as a mental and neural state of readiness, organized along with experience, which has a dynamic or directive effect on an individual's response to objects and situations. According to Rosenberg and Hovland [18], attitude is composed of three main components: cognitive, affective, and behavioral. Fabriagar et al. [19] linked attitude formation to knowledge and memory structures. According to this, to process new knowledge and form a related attitude, we draw on previous experiences and established attitudes. An individual's attitude can be described in terms of the knowledge acquired by a person, the approaches and emotions associated with it, and the patterns of his or her behavior [17–19].

In the present study, we used a qualitative interview method to explore teachers' attitudes toward diabetes care and their interpretations of their roles in supporting children living with T1D in schools and kindergartens. We included teachers of kindergartens, primary schools, and high schools and assistant teachers as well.

In total, 28 participants were working as teachers, and two as assistant teachers. Six of them worked in high schools, nine in primary schools, and 15 worked in kindergartens.

The following research questions guided our study:

1. How can teachers' attitudes toward diabetes care be described within the framework of the three components of attitude?
2. How do teachers perceive their own role in the support of children living with T1D?

## 2. Materials and Methods

### 2.1. Participants

A convenience sampling method was employed to approach the 30 teachers who were included in the study. The participants were reached with the help of school and kindergarten psychologists and a diabetes educator. Other participants were reached through advertisements. Approximately 110 teachers were reached and 30 of them agreed to participate in an interview; hence, the response rate was 27%. The participants did not receive any compensation. The research has been approved by the Human Investigation Review Board at the University of Szeged Albert Szent-Gyorgyi Clinical Center (Ethics Opinion 199/2019-SZTE).

See the participants' characteristics in Table 1. Working directly with a child with diabetes during the course of their work meant that it was in their class or was sometimes under their supervision. Experience with managing diabetes meant that the participant had diabetes (T1D or T2D) or had a spouse living with T2D or a child living with T1D. In terms of educational attainment, all participants held advanced degrees (university or college degrees). We continued to conduct the interviews until data saturation was reached [20].

### 2.2. Interview Questions and Procedure

We started to collect data by conducting focus group interviews. These interviews were led by the second author (a health psychologist researcher) and the first author (a PhD student psychologist) and with the use of a semi-structured questionnaire guide. First, we aimed to explore the participants' shared and specific experiences and opinions on the subject. We also used these interviews to format the interview guide for the individual interviews. We conducted three focus group interviews: one with six participants and two with two participants. In addition, to get more depth and detail on the topics that appeared in the group interviews, 20 individual interviews were conducted. The participants were provided with detailed information about the topic of the interview, the method of transcription, data analysis, and anonymization. Subsequently, they gave written consent to participate. Audio-recording was used to record the interviews, for which consent was also obtained verbally from participants.

### 2.3. Interview Guide

The interview guide consisted of questions within five subjects (see Table 2). We asked the participants about their experiences with diabetes: these questions were about having had people with diabetes in their environment or during their jobs. Within the subject of diabetes-related knowledge, they had to rate their diabetes knowledge from 1 to 10, and we asked them about facts regarding diabetes and where they got their knowledge from. The third subject was about how teachers perceive the options for diabetes care within the institution they work in. We inquired how the care of children with diabetes is managed in the institution and which diabetes management tasks teachers are involved in. Furthermore, we questioned them about the attitudes the peers show toward children with diabetes. The fourth subject focused on the solutions teachers think would be suitable for the problem of the management of diabetes in the institution. Finally, to conclude the interview, we asked them if they wanted to add something to the subjects and how they felt during the interview. See Table 1 for the interview guide used in the interviews.

The individual interviews were led by a PhD student psychologist (first author) and a diabetes educator (third author).

### 2.4. Data Management and Analysis—Thematic Analysis

Each of the interviews was transcribed by the researcher who conducted it. The interviews have been typed verbatim and anonymously (excluding identifiable data). Details about the participants' behaviors were also indicated in transcriptions.

In the present study, a thematic analysis [21, 22] for narrative interviews [23] was applied to the dataset of the interviews. Thematic analysis allows a hybrid analytic approach, i.e., the combination of inductive and deductive reasoning, which we used throughout the entire process of analysis.

Procedures for qualitative analysis outlined by Braun and Clarke [21] and Wu et al. [20] include the following steps:

(1) The authors executing data analysis familiarize themselves with the data by reading and re-reading all of the transcribed interviews.
(2) They then analyzed by the following:
  - generating initial codes.
  - searching for themes—in our case, both inductively and deductively.
  - reviewing themes, and
  - defining and naming themes.

In our study, the first interview was coded together with the second author to create an initial set of codes. Further, in the coding process, 10% of the interviews were independently coded by the first two authors. The occurring differences in coding were discussed until a consensus was reached.

We used the theory of the three components of attitude as a theoretical framework for the coding templates [24], namely the cognitive component, affective component, and behavioral component. An additional theme for the coding of these theoretical groups was the feasibility of diabetes management in schools and kindergartens. Codes and definitions were recorded in a codebook to help the process of analyzing. The first author coded all of the remaining interviews using the codebook. The first two authors held meetings to review codes and generate new emerging ones if it was necessary. The authors agreed on all codes of the transcripts. During the finalization, the investigators organized the codes by relevance to themes aligned with the three components of attitude. All of the codes within the feasibility of diabetes management considering the behaviors of teachers were categorized into the three themes of the components of attitude. See Table 3 for an example of the codes transformed into the final themes and subthemes applied to a short segment of our data.

## 3. Results

**Figure 1:**
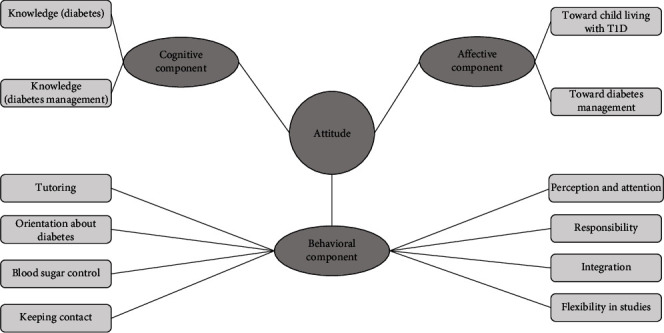
Overview of the subthemes of the components of attitude toward diabetes and its management (circle = main themes, square = subthemes).

An overview of the subthemes of the components of attitude toward diabetes and its management is provided in Figure 1.

### 3.1. Cognitive Component of Attitude toward Diabetes and Its Management

The cognitive component included knowledge about diabetes and its management. Knowledge about diabetes contained both correct and incorrect information about the biological background. Teachers reported the behavioral and psychological signs they observed in children living with T1D (for example, difficulties in concentration during low blood glucose levels). Knowledge about diabetes management included information about the medical treatment of diabetes. The methods of blood sugar control and alternative treatments as additional treatment options for diabetes were mentioned by participants. The majority of teachers in the sample rated their knowledge about diabetes and its management over four (out of 10).

### 3.2. Affective Component of Attitude toward Diabetes and Its Management

The affective component included approach, opinions, and emotions shown toward the child with T1D and its management. We identified cognitive and affective reactions of empathy [25–27]. Cognitive reactions included identifying with the mental perspective of the child with T1D or the parent; affective reactions included expressing vicarious sharing of emotions of the child living with T1D [28]. Most of our participants thought that children with T1D should not be left out of any activities and they should be fully integrated into any community. However, some participants expressed a segregating approach, meaning that children with T1D should go to a separate institution or should do some activities (e.g., sports) separately from their peers. Most of the participants represented the integrating approach and thought that teachers are obliged to learn about diabetes and its management if they must supervise a child with T1D, and they must undertake tasks related to diabetes management.

Some participants expressed that they consider the tasks related to diabetes management as a burden. Others felt that these tasks were not burdensome and easily manageable. The analysis revealed that teachers often experience distress when taking care of a child with T1D. Most commonly, they reported being afraid of the disease itself, of needles or pricking the child with a needle, and of the child falling into a coma. On the other hand, some of the participants reported that they would undertake caring for a child with T1D without any particular anxiety or distress.

### 3.3. Behavioral Component of Attitude toward Diabetes and Its Management

The behavioral component consisted of behaviors teachers exhibit or could be exhibiting for the feasibility of diabetes management in schools and kindergartens. In this case, the subthemes are the specific behaviors that teachers reported performing in order to support the children living with T1D.

The participants pay attention to and perceive different types of diabetes-related changes in the child with T1D, either actively or passively. They actively pay attention and identify symptoms of blood sugar changes. Moreover, they passively let the child with T1D take care of a task related to diabetes management.

Participants expressed that they take responsibility for monitoring the child with T1D or for taking care of diabetes management-related tasks. They may contribute to the integration of the child with T1D by letting them come to extracurricular activities, by informing the child's peers about the disease (explaining diabetes and its treatment), and sensitizing the peers of the child (encouraging them to be more empathic). They also contribute to the integration by guiding peers and modeling how they should treat the child with T1D. Treating children with T1D in the same way as their peers is also a significant part of their contribution to the integration process. School teachers may help children with T1D by being less pressing in their studies. For example, when it comes to physical education, teachers may give easier tasks to them if they have problems with their blood glucose control.

In order to manage diabetes in the institution properly, the participants must keep in touch with the parents of the child with T1D. They also keep in contact with their colleagues. Moreover, one participant mentioned that she would even keep in contact with the medical staff who treat the child with T1D.

Teachers who participated in diabetes care reported that they perform tasks related to blood sugar control or encourage the child to do so (e.g., help with setting the management tool or eating if blood sugar is low and doing some exercise when blood sugar is high). We asked the participants if they would use a glucagon injection in case of an emergency. Most of them said they would, but neither of them had to use it so far.

The participants often look up information about diabetes (mostly on the internet or participate in a training course about diabetes) when they find out that a child with T1D will join their group. They may also gain knowledge about diabetes through experience (personal experience, experience with relatives, or former students with T1D).

The participants also contribute to the children with T1D accepting their disease and being self-sufficient when it comes to diabetes care. They may also increase health awareness in children with T1D and their peers by tutoring them. They consider this as their duty, as they think the nature of their profession requires that they also contribute to the upbringing of the children. See the quotations from each subtheme in Table 4.

In a further analysis, we attempted to determine whether teachers' characteristics (see Table 1 except age) contribute to the responses of distress and burden. We used a binary (0, 1) coding system to quantify the mentions of distress and burden, and a Fisher's exact test was applied to compare the frequencies of these categories alongside the characteristics. None of the results was significant, but it showed a higher frequency of distress within the group having closer experience with managing diabetes on a tendency level (*p*=0.061) (see Table 5).

## 4. Discussion

The aim of the present study was to understand diabetes care in schools and kindergartens from teachers' point of view. We conducted a qualitative study with semi-structured interviews and performed a thematic analysis. The qualitative design allowed the exploration of the underlying mechanisms of teachers' attitudes toward diabetes care. Furthermore, the use of the theoretical framework of the three components of attitude was found suitable for exploring the subject: teachers' attitudes toward diabetes care can be described in terms of knowledge about diabetes and its care, emotions toward diabetes, and behavioral patterns [17–19].

The cognitive component consisted of two main categories: knowledge about diabetes and knowledge about diabetes management. Considering diabetes management, teachers talked about medical treatments, blood sugar control, and alternative treatments. Identifying the behavioral symptoms associated with diabetes is especially important considering the work of teachers, even if they do not specifically help in management. The psychological aspect of diabetes was separated from the behavioral symptoms, as teachers were talking about the psychological traits of children with diabetes.

Within the affective component of attitude (including approach and opinions), we explored categories related to being more open and positive and representing a more integrative approach. Empathy as a positive approach has emerged as a category. Teachers expressed both emotional and cognitive empathy toward children with T1D [25–27]. Teachers working in kindergartens expressed empathy even toward the parents of the children.

As for the negative affections, one of the most frequently mentioned affective components of attitude turned out to be distress. Teachers expressed that they feel uncertain about management tasks (such as blood glucose monitoring and insulin dosing), and they often worry about possible emergency situations (e.g., the pupil falling into a coma) under their supervision. Some teachers also expressed that they consider tasks related to diabetes management as a burden. Distress was less expressed among teachers who had not had closer experience with managing diabetes, and this result was supported by our statistical analysis. None of the other teacher's characteristics (e.g., the age they work with) contributed to the responses of distress and burden.

When reviewing the literature on teaching children with chronic conditions, Hinton and Kirk [29] also found that teachers are afraid of the risks involved with teaching children living with long-term conditions. These fears may originate from the insufficient knowledge of diabetes and its management or from the feeling of being incapable to facilitate its management [30, 31]. In the qualitative study of Boden et al. [15], the consequences of a lack of regulation within schools are reflected within teachers' perceptions of the care of children with diabetes. According to their results, the fear of diabetes care originates from a feeling of incompetence and the high sense of responsibility associated with it. In several other studies, teachers expressed a sense of uncertainty about caring for a child with diabetes due to their inability to deal with an emergency adequately and their concern about possible consequences [32–34].

According to our results, depending on current and previous experiences with diabetes management, teachers witness diabetes care in different ways; some find it scary, stressful, and burdensome, and others might find it easy. Furthermore, the presence of a child with diabetes may bring positive changes in the lives of children in the class, as several teachers have reported that through diabetes, the emphasis on healthy living and acceptance became a regular theme. Further research should be conducted to investigate the impact of the presence of a child with T1D on other children.

The behavioral component consisted of ways teachers contribute to the feasibility of diabetes management in the institution. These behaviors can be categorized into three groups: behavior related to diabetes management tasks, interpersonal relations, and behavior related to the pedagogical profession. Behavior related to management tasks is important, especially in cases where professional support is not available in the institution, as it was mentioned by most of our participants. We also note that it is the same in the majority of schools, as revealed by the Dawn Youth Study [9]. Teachers make a significant contribution to the psychosocial development and integration of the child with T1D by tutoring the children about acceptance (both the child with T1D and their peers) and being self-reliant. Fried et al. [35] also found interpersonal relationships to be a major contributing factor to the way the school supports children with T1D. We found that both informing and sensitizing peers were notable categories, which also highlights the significance and complexity of how teachers can support the integration of children with T1D into their community. Mukherjee et al. [36] also identified that students require support from teachers with being included in school activities, explaining their condition to their peers, and finding support by having someone to talk to about health-related worries.

In general, teachers did not report negative attitudes toward diabetes, as in previous studies [32–34]. Teachers mentioned fear and expressed distress, specifically about the management of diabetes and about taking responsibility for the child's state. However, they were characterized by a sense of empathy, expressed through an integrative approach toward the child with diabetes and peers. Teachers expressed different experiences of the burden of the management of the disease; however, the child's autonomy in the management of diabetes had a positive impact on the experience of the burden. Being open about asking for help may also contribute to positive attitudes toward diabetes care, as teachers who had an open attitude toward diabetes felt that having specific knowledge was not a prerequisite for participating in care. This may refer to the findings of Olson et al. [33], who found that teachers who were less likely to have sufficient knowledge about the disease were less “threatened” by the presence of children with diabetes in their classrooms.

## 5. Conclusions

Children living with T1D may face difficulties in adjusting to their peer community. The role of teachers in facilitating children's integration into the community is significant. Teachers may provide diabetes education to the child's peers, and they may also help children to accept their own condition and manage it more efficiently. Teachers' general empathic approach means that they try to pay attention to the health management of the children, provide some flexibility in the daily routine, communicate with the parent about the child's condition, and carry out tasks around blood glucose control with some help from the parents.

The findings of the present study may guide more detailed examinations of associations between psychological, motivational, and environmental factors in the subject of diabetes management in schools and kindergartens. Professionals training teachers about diabetes management may benefit from our study. Based on our results, more emphasis should be placed on issues that cause distress and burden for teachers (e.g., what to do in case of extremely low blood glucose levels, managing blood glucose control) and the role of teachers in providing emotional support to children with diabetes (e.g., acceptance of T1D, peer sensitization, and education).

## Figures and Tables

**Table 1:** Participant characteristics.

	*N*	(%)
Gender		
Male	4	13.3
Female	26	86.7
Age they work with		
Kindergarten	15	50
Primary or high school	15	50
Working directly with child with T1D		
Yes	19	63.3
No	11	36.7
Experience with managing diabetes		
Yes	7	29.2
No	17	70.8
Received diabetes education		
Yes	15	50
No	15	50

	Mean (SD)	Range

Age in years	42.9 (20)	23–63

**Table 2:** The interview guide.

I. Experiences with diabetes: (1) Do you have/have you ever had diabetes in your close or distant environment? (2) What is your idea of what everyday life is like for someone with diabetes? (3) Is there a child with diabetes in your current or previous work environment?
II. Diabetes-related knowledge: (1) What do you know about diabetes? (2) What types of diabetes do you know? (3) What are the symptoms of diabetes? (4) In kindergarten/school, what are some of the behaviors that can manifest symptoms of diabetes? (5) What causes diabetes? (6) How does diabetes develop? (7) How can diabetes be managed/treated? (8) What treatment tools do you know? (9) Who is involved in the care? (usually for someone with diabetes, not just in school) (10) Where did you get your knowledge about diabetes from (friends, media, education, internet, etc.)
III. Options for diabetes care within school/kindergarten: (1) How is diabetes managed in the kindergarten/school? (2) What daily activities are important in relation to diabetes care in school? (3) How do you see peers relating to a child with diabetes? (4) What specific knowledge/skills does the teacher need if there is a child with diabetes in the group/class? (5) What are the difficulties/barriers to diabetes care within the school? What could be a solution or help to overcome these difficulties? (6) Would you participate in a diabetes education session? (7) Who within the school staff can be affected by diabetes? (8) How are parents contacted about diabetes? (9) What can parents do to facilitate diabetes care for teachers?
IV. Possible solutions to the issue of T1D in schools/kindergartens: (1) What do you think would be an ideal solution to the situation of children living with T1D in school/kindergarten? (2) What realistic/potential/achievable solution do you see? (3) How do you see what you can do to help diabetes management in the school/kindergarten?

**Table 3:** An example of the coding process.

Data extract	Coded for	Final main themes and subthemes
So I have to say that this little girl has such self-discipline in this whole situation that she really sets an example for us. So maybe from a very young age she is obviously involved in this [living with T1D] and gets all the support she needs, but I can really only mention her as an example, that this is not a problem for her, but a natural thing and she is so present in everyday life (II7)	Attitude, affection—empathy—toward child living with T1D	Attitude—affective component—toward child living with T1D

**Table 4:** Quotations of the subthemes.

Themes	Subthemes	Quotations from focus group and individual interviews
Cognitive component	Knowledge about diabetes	“…the pancreas doesn't produce enough insulin, which is needed by the body, so blood sugar levels rise. Well…the symptoms…um…can be drinking a lot, going to the toilet a lot, mouth…breath changes, urine becomes acetous.” (II21)
	Knowledge about diabetes management	“Considering type 1 [diabetes], I understand that insulin needs to be replaced. This can be done via a pen or a pump.” (II11)

Affective component	Toward child living with T1D	“I can see her, poor thing, with the pump and the sensor. Her trousers slip down a little bit and I can see the little red dots on her, and it tugs at my heartstrings that God lets a little 5-year-old face these kind of obstacles.” (II4)
	Toward diabetes management	“I'll tell you that it bothers us quite often. When we're here at work we're studying and concentrating, and P's [the child with T1D] device starts beeping… So, unfortunately we often feel that. 1: It's difficult 2: Tiring 1: A burden!” (FG2)

Behavioral component	Perception and attention	“Here at school, if I see that R's eyes [the child with T1D] become a bit dizzy, I ask him immediately.” (FG1)
	Responsibility	“This child is brought to the school, she spends her time between 8 am till 5 pm here, so during that time I'm responsible for her. And if I'm responsible for her, then my job is to learn the things that are necessary for her.” (FG2)
	Integration	“So that it's very important to talk about it with the other children. Using tales, puppets, we can strengthen the connection [between the child with T1D and his peers]” (II3)
	Flexibility in studies	“It turned out that he had diabetes and he ‘slipped' [failed one school year]. And the question was how we are going to manage to get him to graduation. So, in this case we handled it differently. There wasn't a date for the exam, he could take the exam when he was ready for it.” (FG1)
	Keeping contact	“We were in touch with the parents every single day. They told us how long the child sleeps, how we have to wake him/her up, what size of portions he should eat, etc.” (FG1)
	Blood sugar control	“It happens that when their blood sugar level drops, we give them some cookies, some grape glucose tablets so that they don't start to fall into hypo [hypoglycemia].” (II4)
	Orientation about diabetes	“When I found out that we were going to have [in the group] a little girl like that, I went to the [name of the foundation] Foundation's lecture before she joined the group, so that I could have some theoretical and practical experience of what it entailed.” (II4)
	Tutoring	“Let's think about the situation with glasses. Let's draw a parallel. If a child starts wearing glasses, a smart teacher says: “Wow, you've got such cool glasses” and “Wow it is so good!”. And we prepare the child for this, right? We might even say that the glasses are very fragile, so we have to take good care of them…So, it won't draw too much attention if you introduce it properly.” (II5)

*Note*: Abbreviations: focus groups 1, 2, 3 = FG1, FG2, FG3; individual interviews 1–20 = II 1–20.

**Table 5:** Contingency table of experience with managing diabetes and distress appearing in the answers of the participants.

Experience with managing diabetes	Distress		Total (*n*)
	Yes	No	
Yes	5	2	7
No	4	13	17
Total (*n*)	9	15	24

## Data Availability

The qualitative data used to support the findings of this study have not been made available because we would like to make sure of the anonymity of the participants, and the conducted interviews have been transcribed in Hungarian language.

## Additional Points

*Limitations*. One of the limitations of the present study is that to examine quantitatively whether teachers's characteristics determine the responses our sample was rather small. Another limitation is that we could not understand the perspectives of other parties who are involved in diabetes management in the school and kindergarten settings. Much could be gained from studying the experiences of children, parents, and teachers together.
